# ‘It is relevant, it is useful and we won't be scared to ask': the response of undergraduate dental students to suicide awareness education

**DOI:** 10.1038/s41415-025-8720-5

**Published:** 2025-09-12

**Authors:** Christopher Mackie, Karen McLeod, Conor Davis, Yaa Agyei-Akwa, Aleksandra Omiecinska, Diana Ispas, Lindsay-Jo Sevier-Guy, Abigail Heffernan

**Affiliations:** Dundee Dental Hospital and Research School, 2 Park Place, Dundee, DD1 4HR, United Kingdom

## Abstract

**Introduction** There are over 6,000 deaths by suicide each year in the United Kingdom. National suicide prevention strategies seek to improve the way services assess those who are suicidal. It was highlighted that undergraduate dental students lack confidence when assessing individuals for suicide risk and there is an appetite for education on this topic.

**Aims** To design, deliver and evaluate a brief teaching intervention on suicide awareness for undergraduate dental students using NHS Model for Improvement methodology.

**Methodology** A scoping literature search found no formal framework or evaluation on suicide awareness teaching for undergraduate dental students. Following development and delivery of a pilot teaching intervention, an anonymised questionnaire was distributed with free-text boxes to capture qualitative feedback. Analysis informed subsequent improvement cycles and teaching.

**Results** The vast majority of students felt the teaching was relevant, useful, and reported increased confidence and awareness of how to signpost following disclosures of suicidal ideation. Qualitative feedback was overall very positive, with the importance and universal impact of the topic highlighted. In total, 98% of students felt this topic should continue to be taught at undergraduate level and an appetite was expressed for further teaching.

**Conclusions** There is strong support for further suicide awareness teaching at undergraduate level and scope for a unified approach to suicide prevention teaching in the United Kingdom. Consideration of the lived or living experience of those who have been affected by suicide, alongside input from national experts and charities, will be crucial in the development and delivery of this educational material.

## Key points

- This article explores the complexities and challenges associated with suicide awareness education and details a brief teaching intervention delivered to dental students.
- It shares feedback from undergraduate dental students on the usefulness and relevance of teaching on this topic, alongside qualitative analysis of their responses.
- It was written in conjunction with final-year dental students as they reflected on their role as future dental professionals and the impact of suicide within our society.
- This paper calls for a unified, national approach to suicide awareness education at undergraduate level and shares a number of recommendations for future development.

## Introduction

In 2023, a report published by the University of Manchester highlighted that, between 2010-2020, there were over 6,000 deaths by suicide each year on average in the United Kingdom (UK).^1^ In 2021, suicide rates for England and Wales were 10.7 deaths per 100,000 people,^2^ while in Scotland and Northern Ireland, this statistic was 14.2 and 14.3 deaths per 100,000, respectively.^3^^,^^4^

In Scotland, 73.2% of deaths by suicide were men, and almost half were aged 35-54 at the time of death. Suicide deaths were nearly three times more likely among those from the most socioeconomically deprived areas and nearly 80% had some form of contact with healthcare services in the 12 months before their death.^4^ These services may have included the dental team.

### Suicide, societal stigma and the law

Historically, there has been a complex, reciprocal relationship between suicide and societal stigma,^5^ with many social, economic, psychological and cultural impacts.^6^ The Suicide Act of 1961 decriminalised suicide in England and Wales,^7^ triggering a shift towards more openness and formality around the treatment and recording of attempted suicide across the UK.^8^ In Scotland, while attempting suicide was not a crime itself,^9^ there is evidence to suggest it was still treated as a breach of the peace and investigated until 1958.^10^

Though it hasn't been a crime for half a century, the use of the phrase ‘committed suicide' continues to be (inappropriately) used in common parlance, arguably perpetuating the shame, guilt and stigma people and their families may feel. The Scottish Government publication Creating hope together: Scotland's suicide prevention strategy for 2022-2032 aims to reduce deaths by suicide while tackling the inequalities that contribute to it,^11^ with similar strategies being developed in England,^12^ Wales^13^ and Northern Ireland.^14^

### The impacts of COVID-19

The recent COVID-19 pandemic has been linked with a higher prevalence of suicidal ideation among the general population than was previously reported in the literature,^15^ with young adults, women, those from socially disadvantaged backgrounds and those with pre-existing mental health conditions worst affected.^16^ Several high-profile media campaigns have highlighted the importance of suicide awareness and prevention, including Three Dads Walking,^17^ Emma Webb's Leg on to London^18^ and One Man Walking, A Million Talking.^19^

High-risk groups including clinicians and other frontline healthcare staff may be more prone to the mental health consequences of COVID-19, with risk factors for suicidal ideation including loneliness, low social support, and physical and mental exhaustion.^15^

### Suicide and the dental profession

The dental profession has historically been linked with higher-than-average suicide rates^20^^,^^21^ and, although there is no current consensus on whether this is still the case,^22^ it is clear that occupational stressors and the solitary nature of the role may contribute to higher levels of stress, burnout^23^ and low wellbeing^24^ in the UK.

Between 1995-2011, 77 UK dentists died as a result of suicide, and 17.6% of dentists surveyed on this topic had considered taking their own lives.^25^ Research in Australia has shown that one in six dentists considered taking their own life in the period between 2020-2021 and over 5% of participants made a suicide attempt. This corresponds with UK research that around 10% of dentists considered suicide pre-pandemic.^26^

### Undergraduate dental education on suicide

In 2021, the General Dental Council (GDC) publication Mental health and wellbeing in dentistry: a rapid evidence assessment noted that mental health issues can arise even at undergraduate level and recommended teaching on coping mechanisms, building resilience, and stress management to raise awareness and facilitate early recognition of poor mental health.^27^

Anecdotally, UK undergraduate dental curricula do include recognition of mental health, suicide risk and addictions among the dental team, alongside the impact physical health has on access to and acceptance of dental treatment, but with arguably less formal teaching on suicide awareness relating to patients. Dental professionals have a duty to take a holistic approach to patient care while working within their own mental and physical capabilities.^28^ When recording a patient's medical history, the dental team should enquire about mental health as well as physical conditions.^29^ This should include being able to respond to reports of suicidal ideation.

In April 2023, a British Dental Journal article posed the question: ‘Should suicide risk assessment be embedded in undergraduate dental curricula?'. This highlighted a lack of confidence and uncertainty that may be experienced by dentists and dental students when assessing individuals for suicide risk, and the appetite among undergraduate dental students for education and training in the management of individuals displaying suicidal ideation.^30^

This article opened a discussion among dental educators at a UK university dental school, who opted to widen the discussion to include senior students. It was agreed to use a short section of an in-person seminar on special care dentistry in May 2023, delivered to fourth-year Bachelor of Dental Surgery (BDS4) dental students. This offered a brief overview on the topic of suicide, how to assess a person's suicide risk and respond to suicidal ideation, and where to signpost for help. Educators were cognisant that, though there are a number of tools available to oral health professionals to assess patients at risk of suicide,^31^ there was no universal, agreed-upon standard for assessing suicide risk within a UK dental setting.

## Methodology

A scoping literature search identified evaluation of suicide awareness programmes delivered to undergraduate veterinary students,^32^ and formal teaching,^33^ including peer-to-peer suicide prevention workshops,^34^ for medical students. However, no formal framework or evaluation for teaching on this topic was identified for undergraduate dental students.

**Fig. 1:**
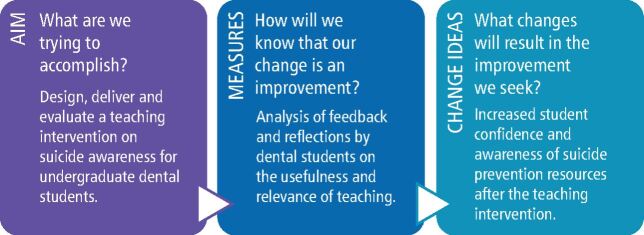
Model for improvement framework questions

**Fig. 2:**
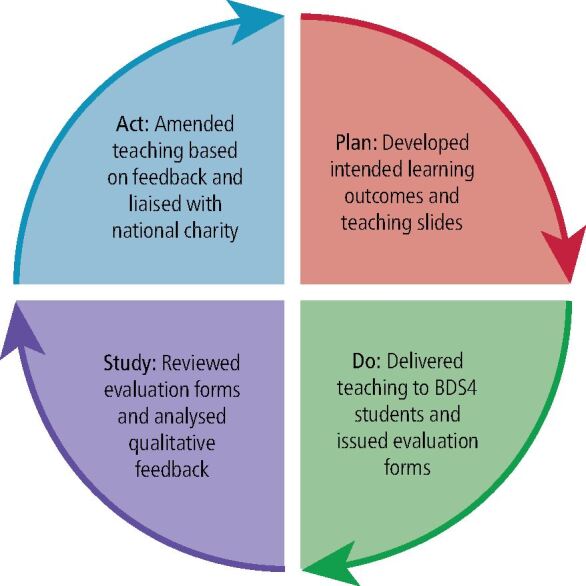
Diagram of Plan, Do, Study, Act cycle used

This article shares the findings of a standalone quality improvement project using Model for Improvement methodology to develop, test and implement changes using a Plan, Do, Study, Act (PDSA) cycle.^35^ The aims statement and initial PDSA cycle are shown in Figure 1 and Figure 2.

To determine the usefulness of this topic in the BDS4 curriculum, a teaching evaluation was conducted. An anonymous paper questionnaire, containing six key questions of interest, was distributed to BDS5 students attending a scheduled, in-person lecture the following academic year. All participating students had received the teaching on suicide in the prior BDS4 module.

The questionnaire contained both quantitative and qualitative components, giving students the opportunity to express their feelings and feedback on the teaching. This aimed to assess whether students felt the teaching was useful and relevant, and whether they felt more empowered to act in cases of patients, friends, or colleagues reporting suicidal ideation. All students participated voluntarily, consented to completion of the questionnaire and were informed before completion that their collective responses would be used in this article.

### Description of teaching

Before this seminar, at a previous in-person lecture, students were informed of the subject matter of the forthcoming teaching, which was integrated into an existing session on mental health conditions. The teaching was designed collaboratively by a consultant in special care dentistry with extensive experience working with patients with serious mental health issues, and a qualified clinical psychologist trained in suicide intervention and prevention, and with experience of managing and training others to manage suicide risk.

Before the session started, it was acknowledged that suicide could be difficult to hear or talk about, and students were reassured that if anyone felt uncomfortable, they were free to leave the session but were encouraged to ‘check in' with a friend or peer later. Students were reminded that at the end of the session, as always, the teaching staff were available to answer any questions privately, or for any students who wished to share comments or ask for help. Reassurance was given that anything discussed would be treated in confidence, unless there was immediate concern for the student's wellbeing.

The section on suicide took the form of a brief teaching intervention and lasted no longer than 20 minutes. It included recent and relevant national and UK statistics, a list of local and national supportive organisations, and how to respond to suicidal ideation and signposting for patients, peers and colleagues. A short anecdote from a retired dental professor actively involved in raising suicide awareness was shared (with permission) with the students and described how a patient had reported that the ‘kind words of their dentist' had encouraged them to seek help and not act on their suicidal ideation.

### Ethics

Input was sought from the regional NHS Research Ethics Committee, who advised that while ethical approval was not required for this evaluation and service improvement project, it was still advisable to register the project with the Clinical Governance and Risk Department. The project was duly registered at both regional and local level within the respective clinical governance departments, and a further communication from the Information Governance Department confirmed that Caldicott Approval was not required for this project.

## Results

**Fig. 3:**
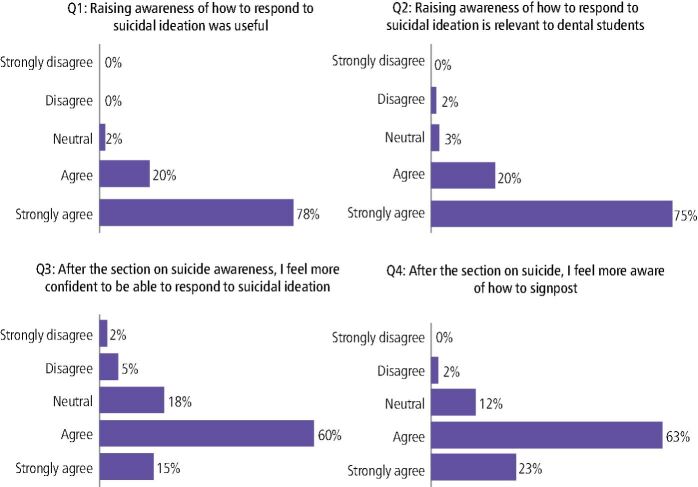
Responses to questions on usefulness, relevance, confidence and awareness of suicidal ideation and signposting

In total, there were 60 respondents (n = 60) from a class size of 69 (87% response rate) and the questions and resulting feedback are detailed in Figure 3.

The vast majority of participants strongly agreed that raising awareness of how to respond to suicidal ideation was useful (78%) and relevant (75%) to dental students, while 63% of respondents agreed that, after the section on suicide, they were more aware of how to signpost to relevant organisations. Although a majority agreed (60%) or strongly agreed (15%) that they felt more confident to respond to suicidal ideation after the training, it must be acknowledged that 25% of respondents gave a neutral or negative response to this statement, and this will be explored in more detail within the discussion.

Overall, these results demonstrate strong support for suicide awareness training among dental students. The majority of participants found the training useful and relevant, and it appeared to increase their confidence in responding to individuals at risk of suicide. Only 15% of respondents had received any prior suicide awareness teaching elsewhere, and the overwhelming consensus among respondents was that we should continue to deliver this training, as 59 out 60 participants agreed (98%). These findings underscore the importance of suicide awareness education in dental undergraduate curricula.

### Qualitative analysis

**Fig. 4:**
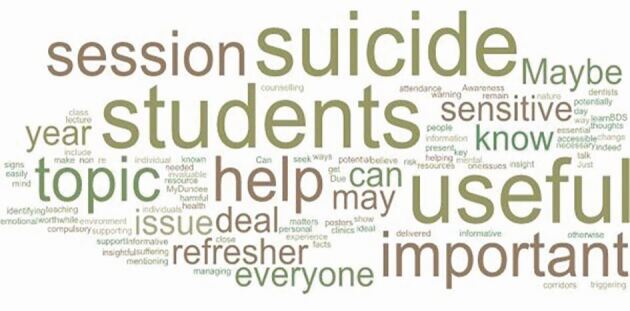
Word cloud of qualitative feedback from students on teaching intervention (note: words that were used more frequently in responses appear larger in the image)

Additionally, students were invited to leave comments on the teaching provided and their responses were used to generate the word cloud in Figure 4.

Inductive thematic analysis of the feedback revealed four emergent themes, supported by the following excerpts of meaning.

#### Relevance and importance of subject matter

**Fig. 5:**
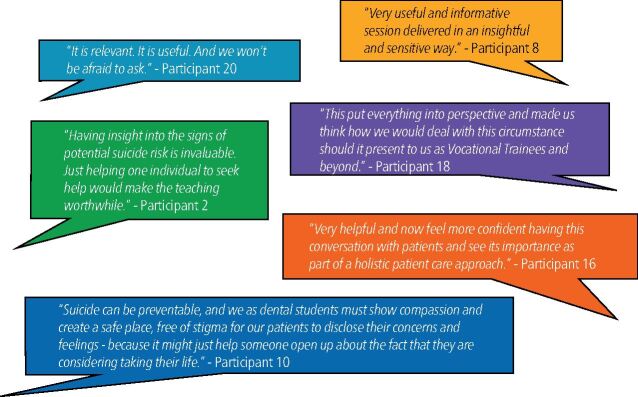
Theme 1 − ‘excerpts of meaning', as communicated by respondents

Participants frequently reported they felt the teaching was useful and important, with many reflecting on the personal and professional impacts of the session and the sensitive manner in which it was delivered. This feedback was overwhelmingly positive (Fig. 5).

#### Subsequent teaching or online follow-up

**Fig. 6:**
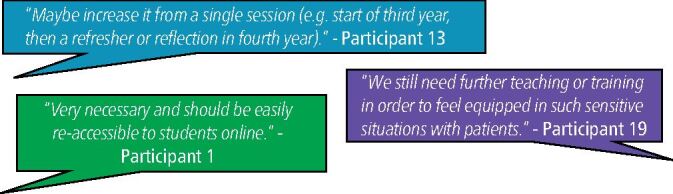
Theme 2 − ‘excerpts of meaning', as communicated by respondents

There was an appetite expressed for further teaching on the topics of suicide awareness and mental health, with students suggesting other topics that might be covered. Participants also suggested accessing material online as an additional resource to tap into post-teaching (Fig. 6).

#### Importance of relevant signposting resources

**Fig. 7:**
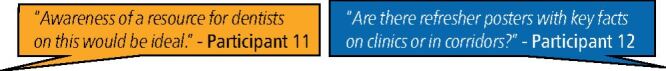
Theme 3 − ‘excerpts of meaning', as communicated by respondents

Students highlighted that signposting resources on undergraduate dental clinics (such as posters or relevant contact information) might facilitate suicide risk assessment and enable signposting to relevant organisations (Fig. 7).

#### Sensitivity of topic and trigger warnings

**Fig. 8:**
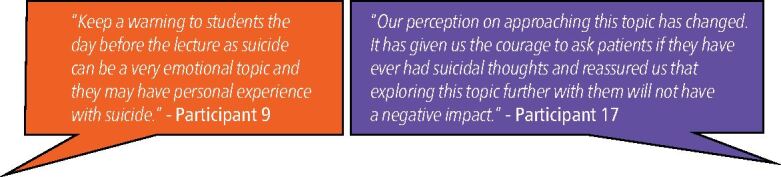
Theme 4 − ‘excerpts of meaning', as communicated by respondents

The emotive and sensitive nature of the topic was highlighted. Students received advanced warning of the material at a prior lecture and at the start of the session, so they would know ahead of time what the session would cover. This ‘trigger warning' was appreciated by participants who felt it was an important aspect of the teaching that should be continued (Fig. 8).

## Discussion and recommendations

It is clear from the feedback that undergraduate dental students felt the suicide awareness teaching was relevant, useful, and increased their confidence in responding and signposting when patients disclose suicidal ideation. A minority (25%) of respondents gave a negative or neutral response to the statement about confidence in responding to suicidal ideation and this merits further exploration.

A systematic review found clinician confidence in suicide risk assessment often sat at over 50% and increased following training - a finding mirrored in this pilot - but that small sample sizes and methodologies which failed to reflect real-life practice meant findings were not generalisable.^36^ Evidence shows that regular, planned and scheduled training for healthcare professionals in suicide risk assessment and management,^37^ complemented by a system of reflective peer review,^38^ increases confidence, and this is one avenue that could be explored in future iterations.

Respondents expressed an appetite for further teaching and supplementary resources to feel better equipped in managing suicidal ideation. It may be that a single seminar is insufficient to foster a high level of confidence for all students, as repetition and revisiting of material is crucial to retention.^39^ The suggestion of a refresher seminar is a reasonable approach to foster a greater sense of confidence among students in responding to suicidal ideation. The teaching itself was received very positively, with an appetite expressed for further sessions within the curriculum; although, a few respondents suggested moving the material online.

### In-person versus online education

Several established and validated suicide awareness and prevention training resources have been identified in the Zero Suicide Alliance's (ZSA's) suicide awareness training,^40^ which is endorsed by NHS Merseyside, and the Grassroots Suicide Prevention ‘safeTALK' module,^41^ which is currently used in postgraduate training of doctors and dentists.

While these modules provide valuable insight into suicide awareness training elsewhere, the funding issues and complexities associated with using such resources for a brief pilot intervention proved prohibitive. While ‘safeTALK' delivers a validated course with trained trainers for sensitive learning, its duration (3.5 hours) and group size (8-30) proved incompatible with our student cohort and timetabling allowances of this pilot study. It does, however, present an opportunity for future collaboration in this area of dental education.

Regarding the online-only format of the ZSA resource, while there is growing appreciation of the benefits of a blended, multimodal education for teaching evidence-based practice^42^ and core knowledge,^43^ the effectiveness^44^ and wellbeing impacts^45^ of in-person teaching must be appreciated. In a global pilot study, undergraduate dental students preferred face-to-face interactions and did not find online learning to be sufficiently interactive.^46^ Respondents themselves highlighted the sensitivity of the topic and the need for continued trigger warnings and, while an online approach may have a protective effect for some and allow students to revisit the material, there are significant safeguarding concerns.

In-person teaching allows for the delivery of a more nuanced seminar whereby the lecturer can assess how the material is being received and answer any questions regarding suicide awareness and signposting. It also encourages students to ‘check-in' with their peers and gives an opportunity for any issues to be addressed swiftly and appropriately. Given the impacts a lack of social support and isolation have on undergraduate students with regards to suicidal ideation,^47^ alongside the sensitive nature of the teaching, it is felt that the current face-to-face seminar is the most appropriate manner in which to deliver this topic.

### Further developments in undergraduate teaching

The decision to deliver this teaching to BDS4 students was chiefly influenced by its perceived compatibility with the topics already covered in the lecture (management of mental health conditions in vulnerable adults) and timetabling constraints. The authors acknowledge that it may have been beneficial to introduce this training earlier in the curriculum to enhance awareness and allow students to build on their existing knowledge as they progress through the course; however, this was not feasible in the context of this pilot project.

Opportunities could be sought to deliver this teaching earlier in the curriculum; however, current constraints on undergraduate timetabling mean it may not be possible to dedicate a full lecture to the topic. Participants did suggest expanding the seminar to incorporate clinical scenarios as examples of best practice and moving the teaching to earlier in the BDS curriculum, so this would provide an opportunity for greater teaching on the topic.

The introduction of relevant signposting resources is another reasonable step following student feedback. It was suggested that a protocol should be implemented for when patients disclose suicidal ideation, and this will likely be developed alongside the teaching and signposting resources. Discussions are currently underway with national charities to develop an *aide memoire*-style resource that can be used on clinics.

A trigger warning will continue to be issued ahead of the session at a prior lecture; however, staff are cognisant that recent research has shown trigger warnings used in isolation can be harmful to students as they may increase distress or exacerbate maladaptive behaviours.^48^ It is therefore important that any future trigger warnings be embedded as part of a broader, trauma-informed and holistic approach to teaching on suicide awareness.

As it was explicitly highlighted as a positive aspect by students, staff will continue to embed it within the teaching but will review and reflect on the appropriateness of this over future PDSA cycles. In keeping with a unified approach to suicide prevention and postvention,^11^ it is also important to consider the lived or living experience of those who have been affected by suicide in developing and delivering this educational material.

### A unified approach to suicide prevention

Feedback indicated an appreciation of the importance and relevance of suicide awareness training and being able to respond to suicidal ideation. Several national and international resources can support dental professionals in their management of mental health emergencies and assisting patients who disclose suicidal ideation. The first, put forward by Yates and Furtado,^49^ includes a flowchart for managing mental health disclosures designed to give dental teams more confidence in these high-pressure situations and ensuring an appropriate standard of support is provided to patients in crisis. Similarly, the FDI digital toolkit, ‘Mental health and well-being in the dental workplace', contains a generic mental health emergency pathway that can be modified to suit the needs of different countries.^50^ Both of these resources can be printed, stored or displayed in dental practices to ensure a unified approach to suicide prevention.

Consultation with national experts and charities is crucial to ensure adherence with national guidelines, and a unified approach across all UK dental schools could prevent duplication or resource wastage when developing a national teaching resource on suicide awareness and risk assessment. Our results appear to indicate strong support for continued teaching on this topic at a local level, and this could potentially be developed into a national resource across other UK dental schools.

Indeed, consideration should be given as to whether this teaching should be delivered to all members of the dental team. A suicide awareness, screening and signposting training pilot for dental staff was evaluated and found to improve self-efficacy and confidence among a cohort of dental care professionals.^51^ This intervention shows early promise and potential to be upscaled at a national level, offering a further opportunity for collaborative education and a unified approach to suicide awareness, prevention and postvention.

### Limitations

As a feasibility and acceptability pilot evaluating suicide awareness and prevention teaching, this quality improvement project shows early promise; however, there are several limitations. The small sample size (n = 60) limits the statistical power and generalisability of the findings;^36^ although, it is still above the median target sample size for UK pilot and feasibility studies.^52^ Additionally, sampling bias cannot be excluded as respondents were from a single year group at a single institution, so the findings may not be predictive of a wider population of undergraduate dental students.

Further bias may be introduced by social desirability, whereby respondents answer in a way that is perceived to be more socially acceptable than their true beliefs; however, the authors sought to limit this by anonymising the questionnaires.^53^ Similarly, framing bias, where the manner in which the teaching is delivered or presented to students influences responses, cannot be excluded.^54^ Finally, there may be resource or logistical challenges associated with scaling this pilot up for a wider audience, but the authors feel strongly this is a timely and important topic that merits further discussion and education, not only at undergraduate level, but for all dental care professionals working in the UK and beyond.

### Recommendations

1. Continue with current undergraduate teaching on suicide awareness
2. Develop and dedicate more time to the topic in future seminars
3. Reflect on the appropriateness of issuing a trigger warning to students in advance of the session due to the sensitive nature of the topic
4. Consider developing and introducing signposting resources and a protocol for when patients disclose suicidal ideation
5. Maintain the current face-to-face approach to teaching but consider making some signposting resources available online
6. Continue to reassess and gather feedback from undergraduate dental students on the teaching provided
7. Explore a unified, national approach to suicide awareness teaching, and begin conversations with national experts and other UK dental schools on this topic.

## Conclusions

Given the increasing prevalence^55^ of suicide^2^ and suicidal ideation,^15^ alongside the growing awareness in our society of its devastating impacts,^19^ teaching on this topic for healthcare staff and students is essential. Preliminary research on the recent COVID-19 pandemic has highlighted its mental health impacts on vulnerable groups^16^ and frontline healthcare staff.^15^ Dentistry has long been associated with substantial levels of stress, exhaustion^23^ and poor mental wellbeing,^24^ and the GDC recommends raising awareness and facilitating early recognition of poor mental health as a part of dental professionals' training.^27^

The positive results of this teaching evaluation highlight the importance and relevance of suicide awareness education, the impact it has on our BDS students and the appetite expressed for further teaching on this topic. We would therefore call upon our colleagues in dental schools across the UK (and beyond) to facilitate this teaching at undergraduate level. Finally, in response to our colleagues who asked whether this should be embedded in undergraduate dental curricula, our response, and that of our students, is an emphatic yes - not only for the benefit of our patients, but for our profession as well.

As one of our undergraduate students commented: ‘it is relevant, it is useful and we won't be scared to ask'.

## Resources

When life is difficult, Samaritans are here - day or night, 365 days a year. You can call them for free on 116 123, email them at jo@samaritans.org, or visit www.samaritans.org to find your nearest branch. Additional resources and helplines are available in Appendix 1.

**Figure:**
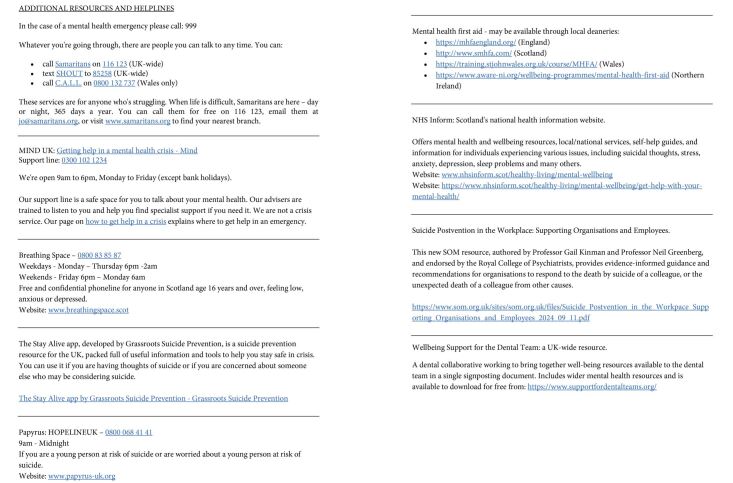
Appendix 1 Additional resources and helplines

## Data Availability

No underlying or extended data were used for this project. All data relevant to this project are included in the article. Hard copies of anonymised questionnaires will be stored securely and then destroyed after one year.
